# Intraseasonal Dynamics and Dominant Sequences in H3N2 Influenza

**DOI:** 10.1371/journal.pone.0008544

**Published:** 2010-01-01

**Authors:** Nicole Creanza, Jason S. Schwarz, Joel E. Cohen

**Affiliations:** 1 Laboratory of Populations, Rockefeller University, New York, New York, United States of America; 2 Earth Institute, Columbia University, New York, New York, United States of America; Smithsonian Institution National Zoological Park, United States of America

## Abstract

Long-term influenza evolution has been well studied, but the patterns of sequence diversity within seasons are less clear. H3N2 influenza genomes sampled from New York State over ten years indicated intraseasonal changes in evolutionary dynamics. Using the mean Hamming distance of a set of amino acid or nucleotide sequences as an indicator of its diversity, we found that influenza sequence diversity was significantly higher during the early epidemic period than later in the influenza season. Diversity was lowest during the peak of the epidemic, most likely due to the high prevalence of a single dominant amino acid sequence or very few dominant sequences during the peak epidemic period, corresponding with rapid expansion of the viral population. The frequency and duration of dominant sequences varied by influenza protein, but all proteins had an abundance of one distinct sequence during the peak epidemic period. In New York State from 1995 to 2005, high sequence diversity during the early epidemic suggested that seasonal antigenic drift could have occurred primarily in this period, followed by a clonal expansion of typically one clade during the peak of the epidemic, possibly indicating a shift to neutral drift or purifying selection.

## Introduction

The three human influenza pandemics of the 20^th^ century originated from avian strains that changed through mutation or reassortment of the RNA segments to produce a virus novel to human immune systems and capable of human-to-human transmission [1]. The recent spread of the avian influenza strain H5N1 and the swine H1N1 strain and the resulting fear that small changes in these viruses could cause a devastating human pandemic have made the goal of understanding influenza evolution timely and important. To date, seasonal influenza has caused cumulatively more deaths than pandemic influenza [2], killing an estimated 30,000 Americans each year [3] and 250,000 to 500,000 worldwide [4]. Over the past decade, many seasonal influenza genomes have been fully sequenced, providing a database for detailed evolutionary studies of the virus.

The wealth of genomic sequence information makes influenza an attractive model for studying sequence change over time in a rapidly evolving system [5], [6]. By a computational analysis of sequence data, we examined patterns of variability in light of the seasonality of flu outbreaks. Influenza A incidence fluctuates seasonally, peaking strongly in the respective winter of temperate regions in each hemisphere. Phylogenetic and coalescent analysis of H3N2 influenza sampled from temperate regions suggested that seasonal epidemics were seeded annually from a reservoir, putatively in East and Southeast Asia [7], [8].

We investigated patterns of influenza sequence diversity in genomic sequence data collected from outpatient visits throughout New York State over ten years (July 1995 to June 2005). We divided each influenza season into early epidemic, peak epidemic, and late epidemic periods and investigated the intraseasonal patterns of sequence variability over time. We found that influenza amino acid and nucleotide diversity were significantly higher during the early epidemic period than during the other periods of the season, consistent with significant non-neutral drift early in the season. Diversity was significantly reduced during the peak epidemic period relative to both the early epidemic and late epidemic periods. Further, this decrease in diversity during the period when influenza infected the most people corresponded to the overrepresentation of one or very few sequences in each season. For each H3N2 protein, many samples from a given season had identical amino acid sequences, particularly during the peak epidemic period. While one distinct amino acid sequence often comprised most of the samples in a season, over time the identity of the dominant sequence changed at different rates depending on the protein. The number of dominant sequences, duration of occurrence, and degree of dominance indicated that the internal proteins evolved differently from antigenic proteins and from one another. This could suggest that the peak of the influenza epidemic is largely driven by the rapid clonal expansion of one group of closely related sequences (many of them identical).

## Methods

### Sequence Alignments

Sequences for each influenza protein were compiled from the NCBI database and aligned by ClustalW using MEGA 4 software [9] (parameters: multiple alignment, Gap opening penalty: 15, Gap extension penalty: 6.66, DNA weight matrix: IUB, Transition Weight: 0.5, Delay divergent cutoff: 30%, Negative matrix: off, ignore predefined gaps). We used 523 H3N2 genomic samples collected from humans in New York State between 1995 and 2005, each containing complete coding DNA and amino acid sequences from all 11 proteins (NCBI Protein search for ‘A/New York H3N2 [protein name]’ for each protein). The sequences were labeled according to the date they were collected and were divided into influenza seasons. Our dataset contained no sequences sampled in June or July of any year, so the boundary between influenza seasons was set at July 1. For example, the 1995 influenza season spanned from July 1995 to June 1996. Each influenza season was separated into three periods, early epidemic, peak epidemic, and late epidemic. For each season, we made a histogram of the number of samples per week and set a threshold at 5% of the total number of sequences in that season. The peak epidemic period was defined as the time from the first week to the last week in which the number of samples exceeded the threshold (inclusive). The early epidemic period lasted from the beginning of the influenza season until the week before the peak epidemic period. The late epidemic period lasted from the week after the peak epidemic period until the end of the season (Figure S1). Seasons that had fewer than 20 samples were not divided into these periods.

### Intraseasonal Analysis

To determine whether there were significant differences in diversity between intraseasonal periods, for each protein we computed the mean Hamming distance of the aligned nucleotide and amino acid sequences in the three periods of each season. The Hamming distance between two sequences of equal length was defined as the number of positions for which the corresponding nucleotides or amino acids were different. To facilitate comparisons between proteins, we calculated the mean pairwise amino acid diversity for each period, defined as the mean number of amino acid differences per site (in other words, the Hamming distance divided by the sequence length). We calculated the standard error by dividing the standard deviation of pairwise sequence diversity measurements within a period by the square root of the number of comparisons within that period.

To compute the mean Hamming distance, we compared the sequence of each isolate to that of every other isolate, finding the number of differences between them, and dividing the total number of differences by the number of pairwise comparisons within that period. We then permuted the order of sequences within each season (such that the sequences were randomly assigned to one of the three periods) and made the same calculations, iterating the randomization 1000 times. The observed mean Hamming distance values were then placed within the distribution of simulated values. If the observed mean Hamming distance was in the bottom 2.5% or the top 2.5% of the distribution, we considered the observed value to be significantly less or significantly more diverse than expected, respectively.

To determine whether there were differences in diversity between the three intraseasonal periods, we counted the instances of significantly high or low observed diversities within each period across seasons and proteins. There were 77 opportunities for a mean Hamming distance to lie outside the center of the distribution of simulated mean Hamming distances (7 seasons with sufficient observations * 11 proteins). The expected number of observations that would lie in the upper 2.5% tail of the distribution of simulated mean Hamming distances by chance alone was 2.5% * 77 = 1.925 and the same for the lower tail. Treating the event of the observed mean Hamming distance lying in the upper 2.5% tail of the distribution as a low-probability independent event, the number of such events should be Poisson distributed with mean 1.925. The 99% critical value for a Poisson distribution with mean 1.925 was 6. There was less than a 1% chance of observing 6 or more instances of significantly low diversity by chance alone, and likewise for significantly high diversity. For each period and each protein, we used a Kruskal-Wallis test to determine whether the diversity of each intraseasonal period was significantly different from that expected by chance alone.

We built phylogenies using the maximum parsimony algorithm under default settings in MEGA4 (Closest neighbor interchange search with a depth of 1 and with the initial trees setting at “random addition trees with 20 replications”). Trees were bootstrapped with 100 replications. We used full-length nucleotide sequences for all trees except when two proteins were encoded by different parts of the same segment, such as M1 and M2, in which case the coding regions were used.

### Dominant Sequence Frequency

For each protein, a large portion of the samples in each season was often composed of identical amino acid sequences. When the amino acid sequences sampled from a season contained identical replicates, we defined the distinct amino acid sequence with the most instances in that season as a dominant sequence and calculated its frequency in every season. For a total of 5 combinations of protein and season, there were exact ties in the frequencies of distinct commonest amino acid sequences; in these cases, no sequence was considered dominant in that season. We then found, for each protein, the number of sequences that met this criterion of being dominant in any season, the average duration of occurrence for the dominant sequences of each protein (measured in number of seasons), and the degree of dominance (calculated by summing the number of samples that were dominant sequences and dividing by the total number of samples in each season). To determine whether the dominant sequences were more prevalent than would be expected by chance, we calculated the proportion of all sequences in each season represented by the dominant sequence(s) for that season and divided that proportion by the expected proportion if all sequences were equally prevalent (found by taking 1 over the total number of distinct sequences in that season). For example, if the dominant sequence represented 40% of all sequences in a given season and there were 20 distinct sequences in that season, 1/20 = 5%, 40%/5% = 8, so the dominant sequence occurred eight times more frequently than predicted by the null hypothesis that all distinct sequences represented an equal proportion of the samples. The minimum and maximum value of this ratio of observed frequency (of each dominant sequence) divided by expected uniform frequency is found in Supplemental Table S1 for each protein in the ‘Minimum Season Dominance’ and ‘Maximum Season Dominance’ columns.

## Results

### Intraseasonal Differences in Diversity

**Figure 1:**
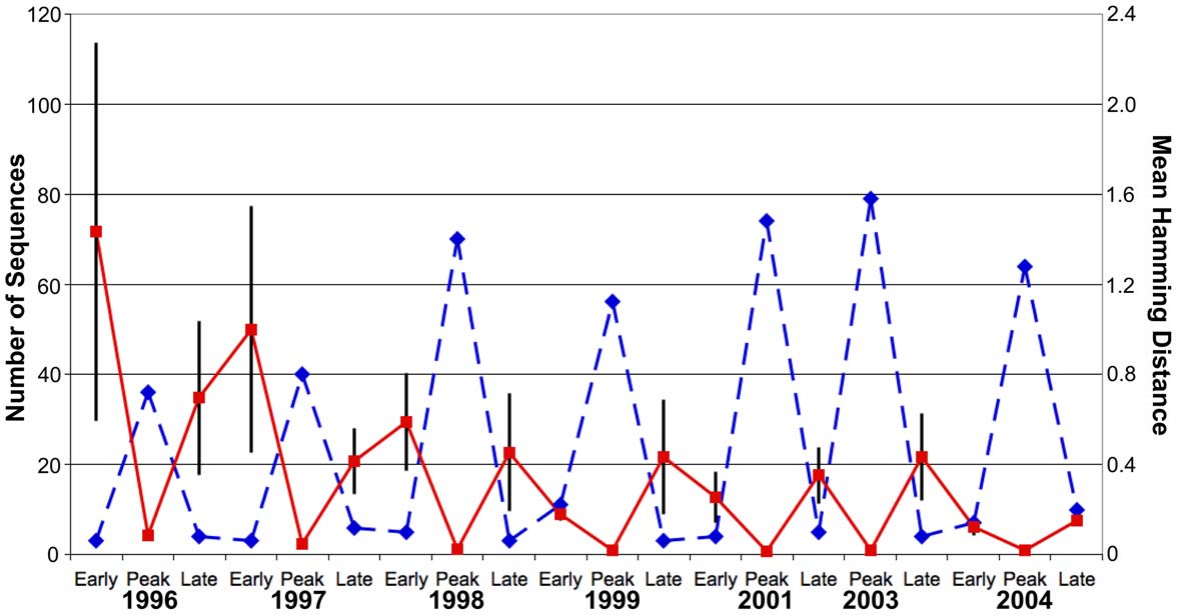
Sequence diversity and number of sequences over time. The mean Hamming distance of the aligned sequences, averaged across proteins, within each period of each season dipped during the peak epidemic periods (solid red line). Error bars indicate mean standard errors of mean sequence diversity across proteins. For every peak period, the standard error of mean Hamming distance was less than 1, too small to be visible here. The non-overlap of the error bars of the peak epidemic periods and the other periods indicates the statistical significance of the reduction in mean Hamming distance during the peak epidemic periods. The peaks in the total number of sequences sampled (dashed blue line) coincided with the dips in mean Hamming distance, suggesting that dominant sequence expansion during the peak epidemic period may have accounted for reduced diversity during the epidemic peak. The scale on the left applies to the number of sequences (dashed blue line).

Peak epidemic periods tended to have lower diversity (mean Hamming distance) than early and late epidemic periods (Figure 1). However, mean Hamming distance was negatively correlated with the number of sequences observed (Figure S2), and peak epidemic periods had much higher numbers of sequences observed than early and late epidemic periods. To control for the effect of sample size on diversity, we randomized the period assignment of each sequence 1000 times (by randomly permuting the dates of each protein's samples within each season) and calculated the mean Hamming distance. We considered a period to have significantly high or low diversity if its mean Hamming distance was in the upper or lower 2.5% of the distribution of randomized Hamming distances for the period. Sequence diversity varied significantly by period within an influenza season (Tables 1 and 2). The early epidemic period tended to be more diverse, and the peak epidemic period tended to be less diverse, than the other periods. For amino acid sequences, high early epidemic diversity and low peak epidemic diversity occurred in the same season in 6 of 11 influenza seasons. For nucleotide sequences, the early epidemic showed significantly higher diversity than expected by chance and the peak epidemic period showed significantly lower diversity than expected. The reduced diversity of the peak epidemic period was consistent with our observation that a single sequence was often dominant during the epidemic. For amino acid sequences, the significant differences in diversity were distributed relatively uniformly across seasons and proteins, suggesting that increased diversity early in the season followed by decreased diversity at the epidemic peak was not a fluke of one season or one protein (Supplemental Tables S2 & S3). The significant differences in diversity were less uniformly distributed across seasons in the nucleotide analysis but were still evenly distributed across proteins (Supplemental Tables S4 & S5).

**Table 1:** Significant differences in amino acid diversity between intraseasonal periods.

	Early epidemic	Peak epidemic	Late epidemic
High diversity	12*	4	3
Low diversity	1	11*	0

The early epidemic period was significantly more diverse than expected relative to the peak epidemic and late epidemic periods. The peak epidemic period was significantly less diverse than expected relative to the early and late epidemic periods. The null hypothesis that the three periods had equal median diversity was rejected by a non-parametric Kruskal-Wallis test (χ^2^ = 48.89, p<0.001). This observation was consistent with the hypothesis that the epidemic peak was associated with the rapid proliferation of a dominant amino acid sequence. An asterisk indicates that the number of high or low diversity events would occur by chance with probability less than 0.01.

**Table 2:** Significant differences in nucleotide diversity between intraseasonal periods.

	Early epidemic	Peak epidemic	Late epidemic
High diversity	7*	2	4
Low diversity	5	15*	0

The nucleotide results mirrored the amino acid results. The early epidemic period was more diverse and the peak epidemic period less diverse than the other periods. The null hypothesis that the three periods had equal median diversity was rejected by the non-parametric Kruskal-Wallis test (χ^2^ = 43.43, p<0.001). These observations were consistent with the results for amino acid sequences, supporting the hypothesis that the epidemic peak was associated with the rapid proliferation of a dominant amino acid sequence. An asterisk indicates that the number of high or low diversity events would occur by chance with probability less than 0.01.

The low diversity and the prevalence of a dominant sequence during the peak epidemic indicated that the peak epidemic was driven by a clonal expansion of very closely related sequences. The high diversity of the early epidemic could be related to the existence of multiple co-circulating clades, or it could indicate some level of non-neutral drift. To investigate further, we looked at phylogenies for each season (Supplemental Figures S3, S4). In most seasons, one particular amino acid sequence dominated during the epidemic peak, and these dominant sequences were clustered in one branch of each phylogeny along with other very closely related but not functionally identical sequences. During the 1996 and 1998 epidemic, multiple clades co-circulated during the epidemic peak, although one was still dominant. Even when there were multiple co-circulating clades of sequences in a season, the sequences within that season remained a consistent Hamming distance from an outgroup (Supplemental Figure S5). In some seasons, such as 2001, the number of samples grew extremely rapidly over time and as a result the early epidemic period was a small proportion of all sequences. Like the HA phylogeny, the phylogenies of other influenza proteins during the 1999 season illustrated the increased diversity of early epidemic sequences (Supplemental Figure S4).

### Dominant Sequences Change over Time

**Figure 2:**
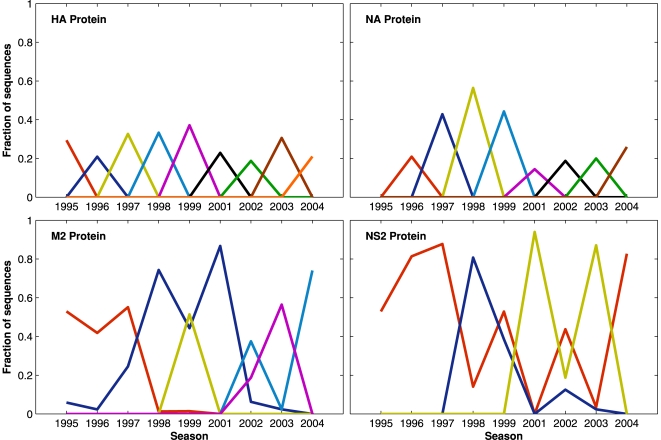
Dominant sequences over time. Different influenza proteins had different temporal patterns of dominant sequences. HA and NA had a different dominant sequence each season, each of which made up a relatively small proportion of samples from that season. Dominant sequences were a larger proportion of all sequences of the internal proteins. The two proteins with the highest proportion of dominant sequences and the longest duration of dominance were NS2 and M2. The fraction of sequences for each dominant sequence changed by season, but nearly all dominant sequences persisted for multiple seasons in these proteins. The x-axis represents influenza season, and the y-axis represents the fraction of all sequences in that season. Each line color represents a distinct dominant sequence, and each occurrence of the dominant sequence had an identical amino acid sequence. E.g., for the HA protein, the dominant sequence represented by the dark blue line was 20% of all HA sequences in the 1996 season, but was absent in 1995 and in 1997 and thereafter.

Across seasons, H3N2 dominant sequences, defined as the distinct amino acid sequence with the most instances in a season, rose and fell at different rates for different proteins. Between 3 and 9 sequences per protein met this criterion for dominance, and the mean duration of these dominant sequences ranged from 1 season for HA and NA to 5 seasons for NS2. When the sequences turned over more slowly, the dominant sequence also tended to make up a larger fraction of the total sequences; the degree of dominance ranged from 0.28 for HA to 0.88 for NS2 (indicating that 88% of NS2 sequences sampled in New York State between 1995 and 2005 were one of only three distinct amino acid sequences). The antigenic proteins had dramatically different seasonal fluctuations in sequence dominance from the internal proteins. Dominant sequences of HA and NA never lasted more than one influenza season (Figure 2).

## Discussion

The database of H3N2 sequences from New York State was a valuable resource for close scrutiny of influenza dynamics. We utilized these data to test predictions about intraseasonal influenza evolution. Influenza nucleotide and protein sequence diversity was highest at the beginning of the epidemic; during the expansion of the epidemic (and of the influenza population), influenza diversity fell significantly (Tables 1 and 2). Based on phylogenies (Supplemental Figures S3-S4) and the prevalence of the dominant sequences (Figure 2), the rapid expansion of influenza during the epidemic peak consisted largely of the clonal expansion of one clade. The evolutionary dynamics behind this clonal expansion were unclear. Mathematical models of evolution within an influenza season predicted differences in sequence evolution under neutral versus non-neutral antigenic drift [10]. When the assumption of neutrality was removed and the host's immunity could affect viral fitness, models indicated that “significant amounts of antigenic drift can occur in the early phases of the epidemic when there are still relatively few infected hosts” and much of the antigenic drift in flu sequences would occur before the peak of the epidemic [10]. It is possible that the increased sequence diversity in the early epidemic was somehow related to non-neutral drift during that period, perhaps across larger populations than we have investigated. Influenza could have shifted between two dominant processes, antigenic drift and purifying selection [11]. In New York State from 1995 to 2005, antigenic drift could have occurred primarily in the early part of each season's epidemic, followed by a clonal expansion in the population of typically one clade. We have no concrete indications that non-neutral drift was occurring early in the epidemic. Analysis of dN/dS ratios between periods was inconclusive, but these small groups of closely related sequences were not particularly amenable to analysis by dN/dS [12]. The finer details of the early period of the epidemic, such as how many distinct sequences are circulating and how fast amino acid changes accumulate, could not be measured with the available data due to the low number of infections and inadequate sampling.

Diversity increased in the early period of the influenza season for most proteins (at least one significant case for all proteins except NP, NS1, and NS2), not merely in the antigenic HA and NA proteins. The decrease in diversity in the peak of the influenza epidemic was also consistent across proteins. It was, perhaps, surprising that these patterns were not limited to the antigenic proteins. The increased diversity early in the epidemic was likely related to the co-circulation of multiple clades, and the reduced diversity during the peak period of the epidemic was related to the clonal expansion of one dominant clade. Differences in diversity were measured within each protein, and the absolute changes were far more dramatic for the antigenic proteins, which had higher baseline diversity, than for the internal proteins, which tended to be more conserved. Understanding influenza evolution on short timescales was inhibited by the low sampling rate of sequences, especially outside the epidemic peak period. By pooling sequences across influenza seasons by protein, we identified significant trends in diversity during a season.

In most seasons, a single protein sequence was dominant. For HA and NA this sequence changed each season. For the internal proteins a single distinct sequence often dominated for several seasons. The prevalence of dominant sequences during the peak of the epidemic was striking and to our knowledge has not been described previously.

Lavenu et al. [13] investigated intraseasonal influenza evolution in the 1999–2000 influenza season in France and found no directional changes in diversity in the course of the epidemic. Because all their samples were from the winter, all were likely from the peak epidemic period. If so, their results were consistent with ours, which revealed very little sequence diversity during the peak of the epidemic. Their data did not allow them to determine whether the virus showed more diversity before the peak epidemic period. In the 2006 influenza season in the United States, Nelson et al. [14] observed one major clade and several small clades of H3N2 sequences, as well as co-circulating H1N1 sequences, suggesting that the pattern of H3N2 evolution might be similar to what we observed even when H3N2 was co-circulating with the H1N1 subtype, a situation not present in the seasons for which we studied intraseasonal dynamics. In our data set, H1N1 was the dominant subtype in the 2000 and 2002 influenza seasons, which contained too few samples for an intraseasonal analysis. We performed a brief analysis of the 2006 season in the United States, which revealed that approximately 25% of H3N2 HA samples in the 2006 season had an identical amino acid sequence, comparable to our average HA dominance of 28.1% in New York State. Several studies, using data that overlapped ours, described the annual New York epidemics as being seeded annually by foreign viruses and suggested that most epidemic diversity was related to the co-circulation of multiple lineages rather than antigenic drift [7], [15]. Our observations cannot distinguish high diversity due to the presence of multiple foreign clades during the early epidemic from high diversity due to non-neutral antigenic drift during the early epidemic. Rambaut *et al.* [7] reported high sequence diversity during the peak epidemic period, an inconsistency with our data that appeared to be related to their diversity metric, which was proportional to effective population size. The prevalence of identical isolates during the epidemic peak, especially considering the high mutation rate and large population size of influenza, could be an indication that selection limited sequence diversity during these epidemic peaks.

Given its few proteins, differences in evolutionary dynamics between those proteins, and wealth of sequence information, influenza offers a unique model of molecular evolution. To study the evolution of this virus more fully, an extended database of complete genomes is required to cover adequately the periods before and after the epidemic peak, surveying influenza patients who might not normally seek treatment, and sampling thoroughly from various geographical regions. This information could further understanding of intraseasonal dynamics, improve vaccine design, clarify the factors affecting virulence, and elucidate the influence of competing influenza strains and subtypes.

## Supporting Information

Figure S1 Method of assigning sequence to period. Method of assigning sequences to early epidemic (red), peak epidemic (green), and late epidemic (blue) periods. For the influenza season lasting from July 1996 to June 1997, the graph shows the fraction of samples from each week of the season. The dashed line is the 5% threshold. Once a single week contained more than 5% of the total number of sequences sampled in a season, the peak epidemic period began. This period lasted until the final time the threshold was crossed.
(0.07 MB TIF)

Figure S2 Mean Hamming distance as a function of the number of sequences. Mean Hamming distance was inversely related to the number of sequences when all early epidemic (red diamonds), peak epidemic (green squares), or late epidemic (blue triangles) periods were considered together. Black x - Simulated mean Hamming values. Each point represents the mean Hamming distance for a single period of a single protein. Regression slopes±standard errors: Data from all periods: −1.10±0.17; Early epidemic: −1.45±0.28; Peak epidemic: 1.63±0.13; Late epidemic: 1.08±0.11; Simulation: −1.06±0.41.
(0.41 MB TIF)

Figure S3 HA phylogenetic trees. Phylogenetic trees of protein HA revealed the clonal nature of the epidemic. Sequences from the early epidemic were labeled in red and those from the late epidemic were labeled in blue. The first sequence sampled from 1995 (labeled in green) was included as an outgroup. Black squares indicate the nucleotide sequences coding for the dominant amino acid sequence. Seasons with very few samples from the early epidemic were seasons where the rate of growth of the numbers of sequences sampled was very rapid.
(2.31 MB PDF)

Figure S4 1999 season phylogenetic trees. The phylogenetic trees of NA, NP, and PB1 for the 1999 season were similar to that of HA, which is identical in Figures S3 and S4. Labeling followed the same pattern as in Figure S3.
(1.20 MB PDF)

Figure S5 Hamming distance over time. The Hamming distance was calculated for each amino acid sequence compared the first sequence sampled in our dataset, at the beginning of the 1995 season (the same outgroup sequence used in phylogenetic analyses) and averaged in two-week intervals over the course of the epidemics studied. The mean Hamming distance was also calculated within each of these two-week intervals; these distances were then plotted over time for HA. The mean Hamming distance compared to the outgroup sequence (red squares) stayed relatively steady during the peak epidemic period of each season, while the mean Hamming distance within two-week intervals (green triangles) showed greater variability in the peak epidemic of most seasons. The source of this variability within two-week intervals was, in most cases, small changes within the strain of sequences that comprised the epidemic peak. In the two seasons when the mean Hamming distance within two-week intervals was consistently high during the epidemic peak, 1996 and 1998, we observed several co-circulating strains, which were more similar to themselves than to each other and persisted throughout the epidemic peak. The number of samples in each two-week interval (blue triangles) was not plotted for seasons with fewer than 20 samples.
(0.07 MB TIF)

Table S1 Dominant sequence characteristics. A dominant sequence was defined as a commonest single distinct amino acid sequence among the samples in a season. Mean duration of occurrence (from first season of occurrence to last season of occurrence, inclusive) is measured in number of seasons. The degree of dominance is the fraction of all sequences that were dominant sequences for each protein. Maximum season dominance and Minimum season dominance are the maximum and minimum, respectively, of the ratio of observed frequency of the dominant sequence for each season divided by expected uniform frequency if all distinct sequences in that season were equally frequent; see text for sample calculation.
(0.05 MB DOC)

Table S2 Incidence of high amino acid diversity by season and protein. Higher-than-expected diversity, consistently seen in the early epidemic period, was fairly evenly distributed across seasons and proteins. Neither a bizarre season nor a single protein drove the increased incidence of high diversity in the early epidemic period. The early epidemic period in the 2003 season was removed due to small sample size.
(0.07 MB DOC)

Table S3 Incidence of low amino acid diversity by season and protein. Lower-than-expected diversity, consistently seen in the peak epidemic period, was also fairly evenly distributed across seasons and proteins, like higher-than-expected diversity.
(0.07 MB DOC)

Table S4 Incidence of high nucleotide diversity by season and protein. Higher-than-expected nucleotide diversity, consistently seen in the early epidemic period, was less evenly distributed across seasons than the amino acid diversity, but was still broadly distributed across proteins. These results are consistent with the amino acid diversity levels in Table S2. The early epidemic period in the 2003 season was removed due to small sample size.
(0.07 MB DOC)

Table S5 Incidence of low nucleotide diversity by season and protein. Lower-than-expected nucleotide diversity, consistently seen in the peak epidemic period, was fairly evenly distributed across seasons and proteins, much like the amino acid diversity.
(0.07 MB DOC)
