# Supplementary figures and images for: Intraseasonal Dynamics and Dominant Sequences in H3N2 Influenza
Source: PLoS One. 2010 Jan 1;5(1):e8544. doi: 10.1371/journal.pone.0008544 (PMC2796395; doi:10.1371/journal.pone.0008544)

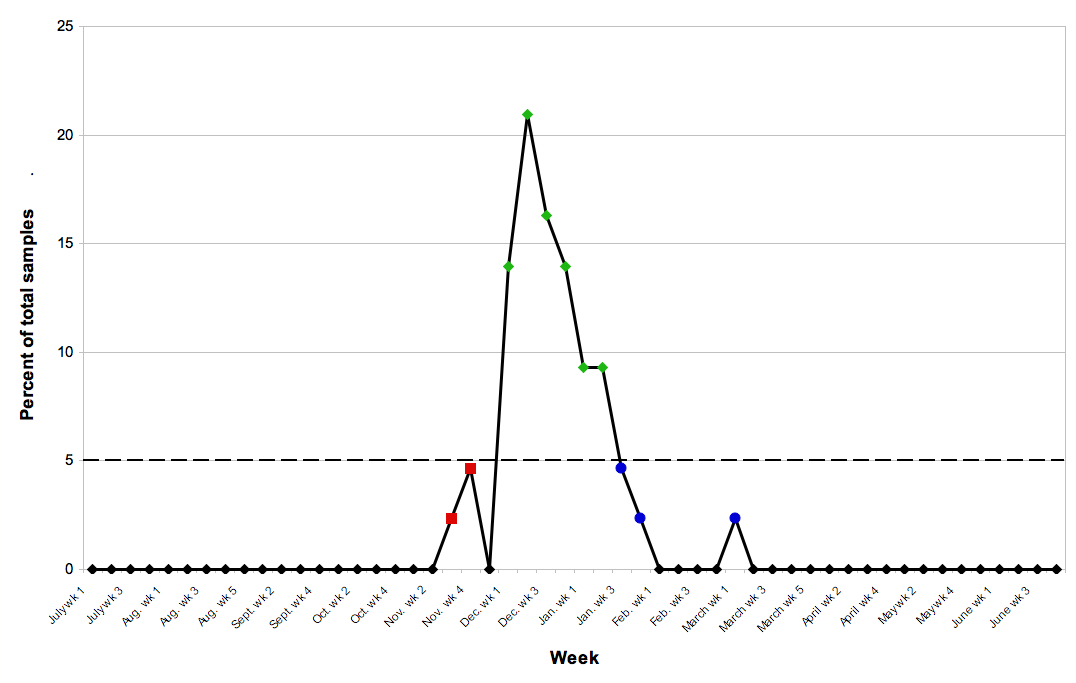

Supplement: Figure S1 — Method of assigning sequence to period. Method of assigning sequences to early epidemic (red), peak epidemic (green), and late epidemic (blue) periods. For the influenza season lasting from July 1996 to June 1997, the graph shows the fraction of samples from each week of the season. The dashed line is the 5% threshold. Once a single week contained more than 5% of the total number of sequences sampled in a season, the peak epidemic period began. This period lasted until the final time the threshold was crossed. (0.07 MB TIF) [file pone.0008544.s001.tif]

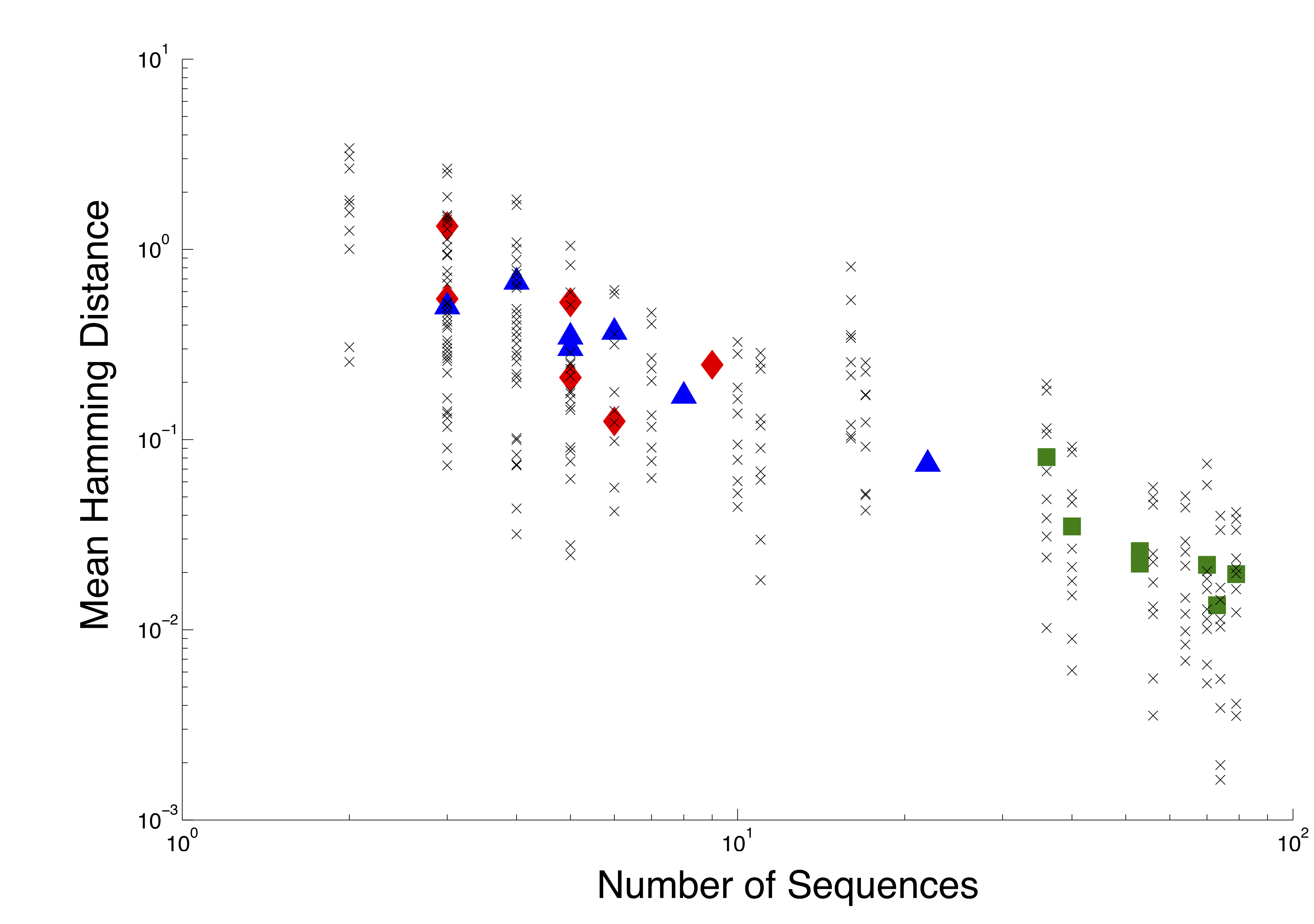

Supplement: Figure S2 — Mean Hamming distance as a function of the number of sequences. Mean Hamming distance was inversely related to the number of sequences when all early epidemic (red diamonds), peak epidemic (green squares), or late epidemic (blue triangles) periods were considered together. Black x - Simulated mean Hamming values. Each point represents the mean Hamming distance for a single period of a single protein. Regression slopes±standard errors: Data from all periods: −1.10±0.17; Early epidemic: −1.45±0.28; Peak epidemic: 1.63±0.13; Late epidemic: 1.08±0.11; Simulation: −1.06±0.41. (0.41 MB TIF) [file pone.0008544.s002.tif]

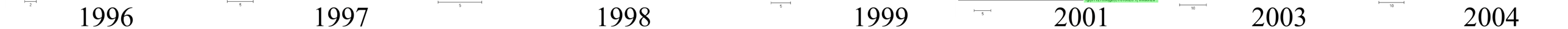

Supplement: Figure S3 — HA phylogenetic trees. Phylogenetic trees of protein HA revealed the clonal nature of the epidemic. Sequences from the early epidemic were labeled in red and those from the late epidemic were labeled in blue. The first sequence sampled from 1995 (labeled in green) was included as an outgroup. Black squares indicate the nucleotide sequences coding for the dominant amino acid sequence. Seasons with very few samples from the early epidemic were seasons where the rate of growth of the numbers of sequences sampled was very rapid. (2.31 MB PDF) [file pone.0008544.s003.pdf]

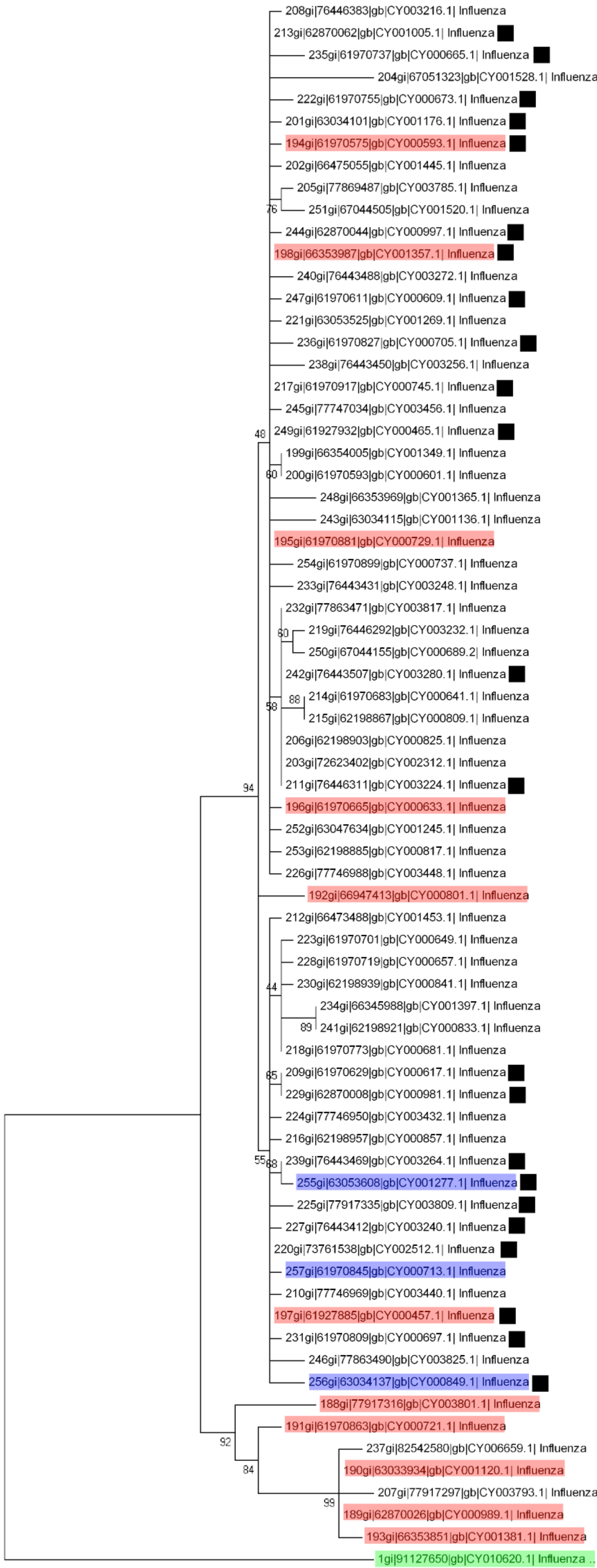

HA

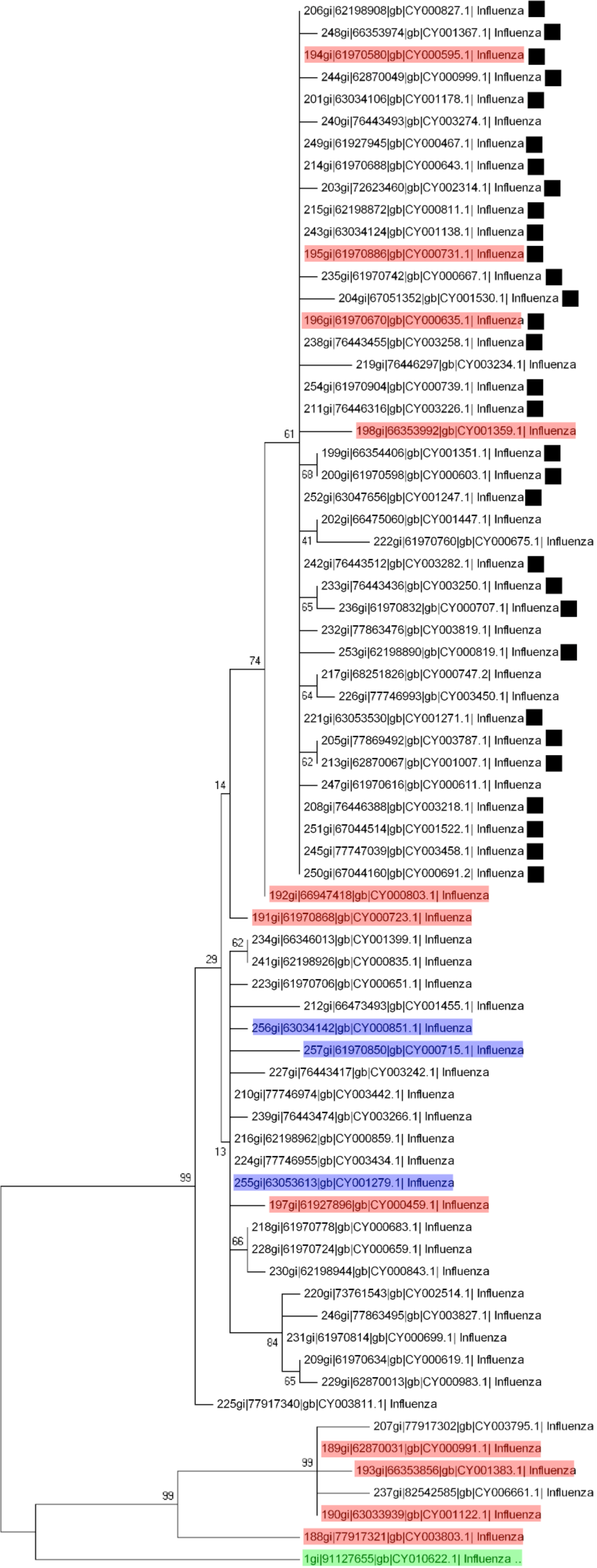

NA

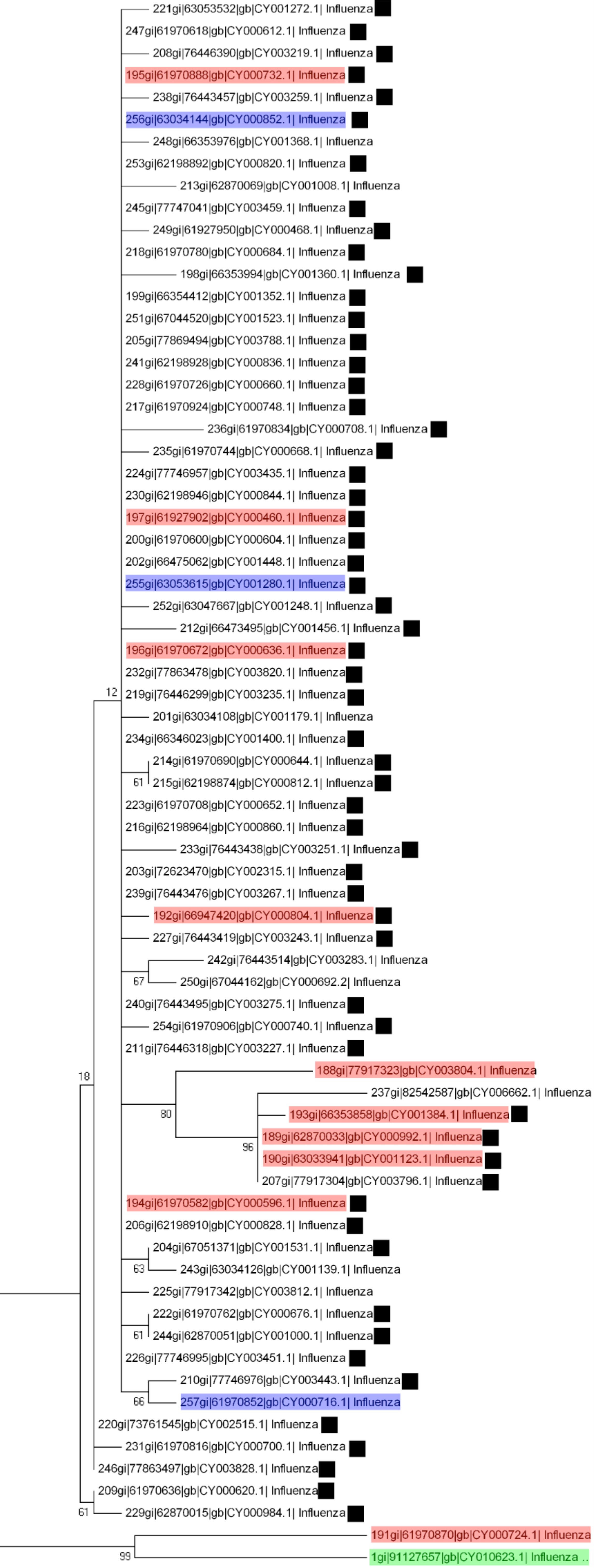

NP

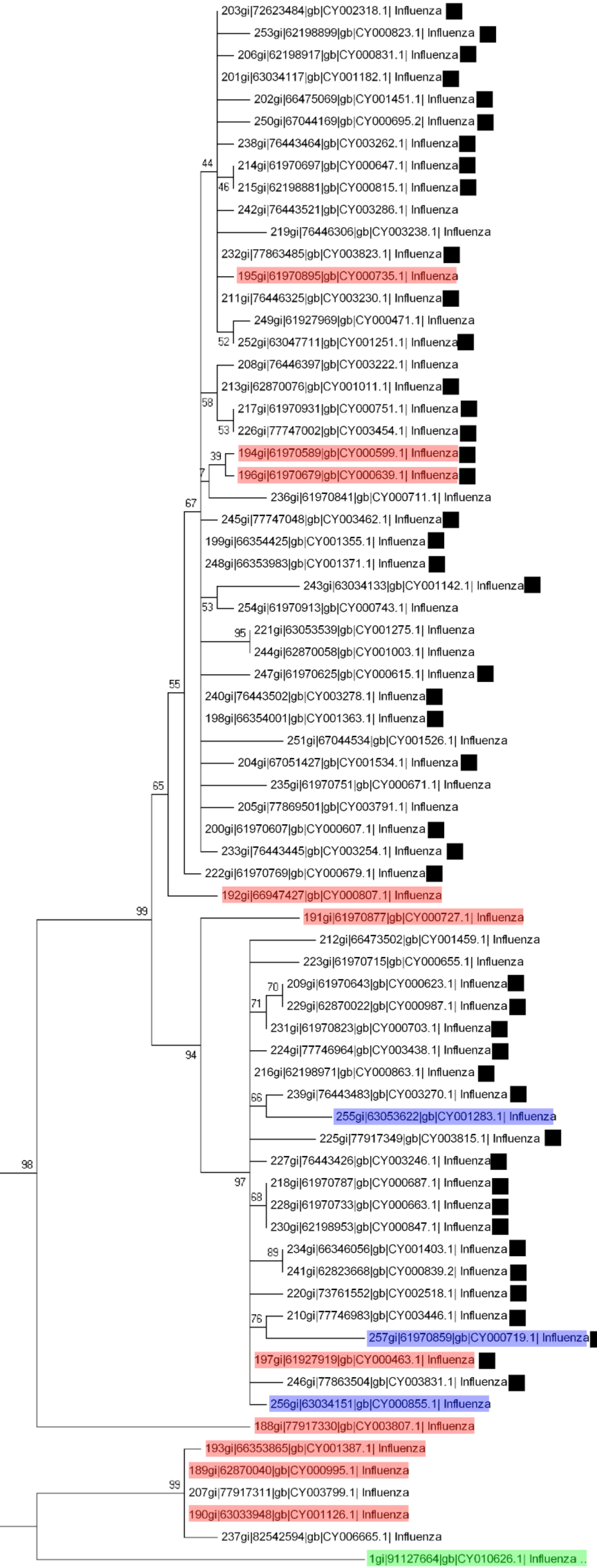

PB1

Supplement: Figure S4 — 1999 season phylogenetic trees. The phylogenetic trees of NA, NP, and PB1 for the 1999 season were similar to that of HA, which is identical in Figures S3 and S4. Labeling followed the same pattern as in Figure S3. (1.20 MB PDF) [file pone.0008544.s004.pdf]

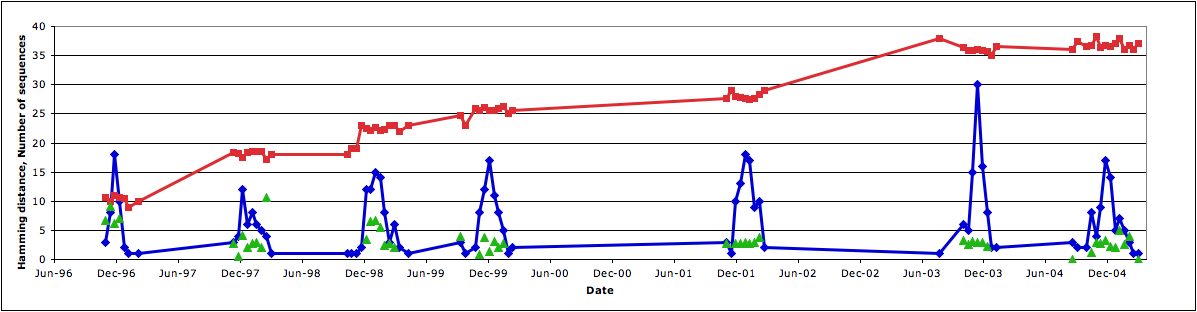

Supplement: Figure S5 — Hamming distance over time. The Hamming distance was calculated for each amino acid sequence compared the first sequence sampled in our dataset, at the beginning of the 1995 season (the same outgroup sequence used in phylogenetic analyses) and averaged in two-week intervals over the course of the epidemics studied. The mean Hamming distance was also calculated within each of these two-week intervals; these distances were then plotted over time for HA. The mean Hamming distance compared to the outgroup sequence (red squares) stayed relatively steady during the peak epidemic period of each season, while the mean Hamming distance within two-week intervals (green triangles) showed greater variability in the peak epidemic of most seasons. The source of this variability within two-week intervals was, in most cases, small changes within the strain of sequences that comprised the epidemic peak. In the two seasons when the mean Hamming distance within two-week intervals was consistently high during the epidemic peak, 1996 and 1998, we observed several co-circulating strains, which were more similar to themselves than to each other and persisted throughout the epidemic peak. The number of samples in each two-week interval (blue triangles) was not plotted for seasons with fewer than 20 samples. (0.07 MB TIF) [file pone.0008544.s005.tif]
