# Supplementary material for: Intraseasonal Dynamics and Dominant Sequences in H3N2 Influenza
Source: PLoS One. 2010 Jan 1;5(1):e8544. doi: 10.1371/journal.pone.0008544 (PMC2796395; doi:10.1371/journal.pone.0008544)
Supplement: Table S1 — Dominant sequence characteristics. A dominant sequence was defined as a commonest single distinct amino acid sequence among the samples in a season. Mean duration of occurrence (from first season of occurrence to last season of occurrence, inclusive) is measured in number of seasons. The degree of dominance is the fraction of all sequences that were dominant sequences for each protein. Maximum season dominance and Minimum season dominance are the maximum and minimum, respectively, of the ratio of observed frequency of the dominant sequence for each season divided by expected uniform frequency if all distinct sequences in that season were equally frequent; see text for sample calculation. (0.05 MB DOC) [file pone.0008544.s006.doc]

**Supplemental Table S1.** Dominant sequence characteristics

| Protein | Number of dominant sequences | Duration of occurrence | Degree of dominance | Maximum season dominance | Minimum season dominance |
| --- | --- | --- | --- | --- | --- |
| HA | 9 | 1 | 0.281 | 14.00 | 2.25 |
| M1 | 4 | 2.5 | 0.826 | 6.26 | 2.00 |
| M2 | 5 | 3.8 | 0.744 | 12.64 | 2.57 |
| NA | 8 | 1 | 0.302 | 12.40 | 1.80 |
| NP | 5 | 2.2 | 0.660 | 11.47 | 1.76 |
| NS1 | 8 | 1.875 | 0.627 | 13.33 | 2.35 |
| NS2 | 3 | 5 | 0.881 | 9.93 | 2.12 |
| PA | 5 | 1.8 | 0.407 | 14.96 | 2.25 |
| PB1 | 5 | 2 | 0.518 | 15.52 | 2.25 |
| PB2 | 8 | 1.75 | 0.539 | 8.60 | 1.76 |
| PB1F2 | 7 | 1.857 | 0.541 | 18.02 | 2.47 |
