# Supplementary material for: Intraseasonal Dynamics and Dominant Sequences in H3N2 Influenza
Source: PLoS One. 2010 Jan 1;5(1):e8544. doi: 10.1371/journal.pone.0008544 (PMC2796395; doi:10.1371/journal.pone.0008544)
Supplement: Table S2 — Incidence of high amino acid diversity by season and protein. Higher-than-expected diversity, consistently seen in the early epidemic period, was fairly evenly distributed across seasons and proteins. Neither a bizarre season nor a single protein drove the increased incidence of high diversity in the early epidemic period. The early epidemic period in the 2003 season was removed due to small sample size. (0.07 MB DOC) [file pone.0008544.s007.doc]

**Supplemental** **Table S2.** Incidence of high amino acid diversity by season, period and protein

| Season | Early epidemic | Peak epidemic | Late epidemic |  | Total |
| --- | --- | --- | --- | --- | --- |
| 1996 | 2 | 2 | 0 |  | 4 |
| 1997 | 1 | 0 | 0 |  | 1 |
| 1998 | 4 | 0 | 1 |  | 5 |
| 1999 | 3 | 1 | 0 |  | 4 |
| 2001 | 2 | 1 | 1 |  | 4 |
| 2003 |  | 0 | 0 |  | 0 |
| 2004 | 0 | 0 | 1 |  | 1 |

| Protein | Early epidemic | Peak epidemic | Late epidemic |  | Total |
| --- | --- | --- | --- | --- | --- |
| HA | 1 | 0 | 0 |  | 1 |
| M1 | 2 | 1 | 1 |  | 4 |
| M2 | 1 | 0 | 0 |  | 1 |
| NA | 2 | 0 | 0 |  | 2 |
| NP | 0 | 0 | 0 |  | 0 |
| NS1 | 0 | 1 | 1 |  | 2 |
| NS2 | 0 | 2 | 0 |  | 2 |
| PA | 1 | 0 | 0 |  | 1 |
| PB1 | 1 | 0 | 0 |  | 1 |
| PB2 | 1 | 0 | 0 |  | 1 |
| PB1F2 | 3 | 0 | 1 |  | 4 |
