# Supplementary material for: Intraseasonal Dynamics and Dominant Sequences in H3N2 Influenza
Source: PLoS One. 2010 Jan 1;5(1):e8544. doi: 10.1371/journal.pone.0008544 (PMC2796395; doi:10.1371/journal.pone.0008544)
Supplement: Table S3 — Incidence of low amino acid diversity by season and protein. Lower-than-expected diversity, consistently seen in the peak epidemic period, was also fairly evenly distributed across seasons and proteins, like higher-than-expected diversity. (0.07 MB DOC) [file pone.0008544.s008.doc]

**Supplemental Table S3.** Incidence of low amino acid diversity by season, period and protein

| Season | Early epidemic | Peak epidemic | Late epidemic |  | Total |
| --- | --- | --- | --- | --- | --- |
| 1996 | 0 | 0 | 0 |  | 0 |
| 1997 | 0 | 0 | 0 |  | 0 |
| 1998 | 0 | 2 | 0 |  | 2 |
| 1999 | 0 | 4 | 0 |  | 4 |
| 2001 | 0 | 2 | 0 |  | 2 |
| 2003 |  | 2 | 0 |  | 2 |
| 2004 | 1 | 1 | 0 |  | 2 |

| Protein | Early epidemic | Peak epidemic | Late epidemic |  | Total |
| --- | --- | --- | --- | --- | --- |
| HA | 1 | 1 | 0 |  | 2 |
| M1 | 0 | 1 | 0 |  | 1 |
| M2 | 0 | 1 | 0 |  | 1 |
| NA | 0 | 1 | 0 |  | 1 |
| NP | 0 | 0 | 0 |  | 0 |
| NS1 | 0 | 1 | 0 |  | 1 |
| NS2 | 0 | 0 | 0 |  | 0 |
| PA | 0 | 2 | 0 |  | 2 |
| PB1 | 0 | 1 | 0 |  | 1 |
| PB2 | 0 | 2 | 0 |  | 2 |
| PB1F2 | 0 | 1 | 0 |  | 1 |
