# Supplementary material for: Intraseasonal Dynamics and Dominant Sequences in H3N2 Influenza
Source: PLoS One. 2010 Jan 1;5(1):e8544. doi: 10.1371/journal.pone.0008544 (PMC2796395; doi:10.1371/journal.pone.0008544)
Supplement: Table S4 — Incidence of high nucleotide diversity by season and protein. Higher-than-expected nucleotide diversity, consistently seen in the early epidemic period, was less evenly distributed across seasons than the amino acid diversity, but was still broadly distributed across proteins. These results are consistent with the amino acid diversity levels in Table S2. The early epidemic period in the 2003 season was removed due to small sample size. (0.07 MB DOC) [file pone.0008544.s009.doc]

**Supplemental Table S4.** Incidence of high nucleotide diversity by season, period and protein

| Season | Early epidemic | Peak epidemic | Late epidemic |  | Total |
| --- | --- | --- | --- | --- | --- |
| 1996 | 0 | 0 | 0 |  | 0 |
| 1997 | 0 | 0 | 0 |  | 0 |
| 1998 | 2 | 0 | 0 |  | 2 |
| 1999 | 4 | 0 | 0 |  | 4 |
| 2001 | 1 | 0 | 1 |  | 2 |
| 2003 | 0 | 0 | 0 |  | 0 |
| 2004 | 0 | 2 | 3 |  | 5 |

| Protein | Early epidemic | Peak epidemic | Late epidemic |  | Total |
| --- | --- | --- | --- | --- | --- |
| HA | 1 | 0 | 1 |  | 2 |
| M1 | 1 | 1 | 0 |  | 2 |
| M2 | 1 | 0 | 0 |  | 1 |
| NA | 1 | 1 | 0 |  | 2 |
| NP | 1 | 0 | 1 |  | 2 |
| NS1 | 0 | 0 | 0 |  | 0 |
| NS2 | 0 | 0 | 0 |  | 0 |
| PA | 1 | 0 | 0 |  | 1 |
| PB1 | 1 | 0 | 1 |  | 2 |
| PB2 | 0 | 0 | 0 |  | 0 |
| PB1F2 | 0 | 0 | 1 |  | 1 |
