# Supplementary material for: Intraseasonal Dynamics and Dominant Sequences in H3N2 Influenza
Source: PLoS One. 2010 Jan 1;5(1):e8544. doi: 10.1371/journal.pone.0008544 (PMC2796395; doi:10.1371/journal.pone.0008544)
Supplement: Table S5 — Incidence of low nucleotide diversity by season and protein. Lower-than-expected nucleotide diversity, consistently seen in the peak epidemic period, was fairly evenly distributed across seasons and proteins, much like the amino acid diversity. (0.07 MB DOC) [file pone.0008544.s010.doc]

**Supplemental** **Table S5.** Incidence of low nucleotide diversity by season, period and protein

| Season | Early epidemic | Peak epidemic | Late epidemic |  | Total |
| --- | --- | --- | --- | --- | --- |
| 1996 | 0 | 1 | 0 |  | 1 |
| 1997 | 0 | 0 | 0 |  | 0 |
| 1998 | 0 | 1 | 0 |  | 1 |
| 1999 | 0 | 8 | 0 |  | 8 |
| 2001 | 0 | 2 | 0 |  | 2 |
| 2003 | 0 | 3 | 0 |  | 3 |
| 2004 | 5 | 0 | 0 |  | 5 |

| Protein | Early epidemic | Peak epidemic | Late epidemic |  | Total |
| --- | --- | --- | --- | --- | --- |
| HA | 0 | 3 | 0 |  | 3 |
| M1 | 0 | 1 | 0 |  | 1 |
| M2 | 0 | 2 | 0 |  | 2 |
| NA | 1 | 1 | 0 |  | 2 |
| NP | 0 | 2 | 0 |  | 2 |
| NS1 | 1 | 1 | 0 |  | 2 |
| NS2 | 0 | 1 | 0 |  | 1 |
| PA | 1 | 2 | 0 |  | 3 |
| PB1 | 1 | 1 | 0 |  | 2 |
| PB2 | 0 | 0 | 0 |  | 0 |
| PB1F2 | 1 | 1 | 0 |  | 2 |
